# Effectiveness and safety of non-vitamin K direct oral anticoagulants in atrial fibrillation patients with bioprosthetic valve

**DOI:** 10.1371/journal.pone.0268113

**Published:** 2022-06-14

**Authors:** Inki Moon, Tae-Hwa Go, Jang Young Kim, Dae Ryong Kang, Suk Ho Sohn, Hyun-Jung Lee, Jae-Woong Choi, Jun-Bean Park, Ho-Young Hwang, Hyung-Kwan Kim, Yong-Jin Kim, Kyung-Hwan Kim, Seung-Pyo Lee

**Affiliations:** 1 Division of Cardiology, Department of Internal Medicine, Soonchunhyang University Bucheon Hospital, Bucheon, Republic of Korea; 2 Department of Biostatistics, Yonsei University Wonju College of Medicine, Wonju, Republic of Korea; 3 Department of Internal Medicine, Yonsei University Wonju College of Medicine, Wonju, Republic of Korea; 4 Department of Precision Medicine, Yonsei University Wonju College of Medicine, Wonju, Republic of Korea; 5 Department of Cardiovascular Thoracic Surgery, Seoul National University Hospital, Seoul, Republic of Korea; 6 Department of Internal Medicine, Seoul National University Hospital, Seoul, Republic of Korea; University of Palermo, ITALY

## Abstract

**Background:**

Non-vitamin K direct oral anticoagulant (DOAC) is effective for prevention of embolic events in nonvalvular atrial fibrillation (AF) patients. However, the effectiveness and safety of DOAC in AF patients who have bioprosthetic heart valve (BPHV) is largely unknown.

**Methods:**

We retrospectively identified patients with AF and BPHV, using the diagnostic code and medical device and surgery information from the Korean National Health Insurance Service database, between 2013 and 2018. A 1:2 propensity score-matched cohort (n = 724 taking warfarin; n = 362 taking DOAC) was constructed and analyzed for the primary clinical outcome, a composite of ischemic stroke and systemic embolism. Important secondary outcomes included major bleeding, all-cause death, and the net clinical outcome, defined as a composite of all embolic events, major bleeding, and death.

**Results:**

The mean age was 78.9±6.8 years old, and 45% (n = 489) were male. The mean CHA2DS2-VASc score was 4.7±1.4. DOAC was non-inferior to warfarin for preventing ischemic stroke and systemic embolism (hazard ratio [HR] 1.14, 95% confidence interval [CI] 0.56–2.34), major bleeding (HR 0.80, 95% CI 0.32–2.03) and all-cause death (HR 1.09, 95% CI 0.73–1.63). As for the net clinical outcome, DOAC was also similar to warfarin (HR 1.06, 95% CI 0.76–1.47). These outcomes were not different in various subgroups analyzed.

**Conclusion:**

In this nationwide Korean AF population with a BPHV, DOAC was at least as effective and safe as warfarin for the prevention of systemic embolic events. These results suggest that DOAC may be an excellent alternative to warfarin in AF patients with BPHV.

## Introduction

Atrial fibrillation (AF) is an independent risk factor for ischemic stroke and systemic embolism (ISSE). Therefore, the prevention of ISSE is a major objective of treatment in AF patients [1]. After several pivotal randomized clinical trials that evaluated the efficacy and safety of non-vitamin K direct oral anticoagulants (DOAC) in AF [2–5], DOAC has gained popularity for ISSE prevention in AF patients. However, these trials excluded those with significant valvular heart disease, and there is limited evidence on the use of DOAC for AF patients with various comorbidities [1].

In recent expert consensus statements [6], the use of DOAC has been recommended in AF patients with valvular heart disease based on post-hoc analysis of randomized clinical trials [7], and retrospective studies [8,9]. However, again, there are exceptions such as those with significant mitral stenosis and mechanical heart valves. Although patients with bioprosthetic heart valve (BPHV) are also possible candidates of DOAC use, the evidence supporting this is limited–an observational report of similar thromboembolic risk in those with BPHV compared to those with nonvalvular AF [10], and small-sized post-hoc analysis of randomized trials and pilot studies in these specific patients [11]. Recently, a randomized trial showed the non-inferiority of rivaroxaban compared to warfarin in this group [12]; however, the evidence from real-world practice is still short.

In this analysis, we hypothesized that DOAC would be as effective as warfarin for the prevention of ISSE in patients with AF and BPHV. The objective of this study was to investigate the effectiveness and safety of DOAC compared to warfarin, specifically in AF patients with concomitant BPHV, using a Korean nationwide cohort.

## Methods

This study analysis plan was approved by the Seoul National University Hospital Institutional Review Board (E-1903-048-1016). This research was done without patient involvement.

### Data source

We used the national claims data established by the National Health Insurance Service of Korea for the current retrospective observational analysis. The database has access to all medical records of the entire nationwide Korean population covered by the obligatory National Health Insurance Service and Medical Aid program. Each patients’ sociodemographic information, diagnoses, procedures and surgery, and prescription records are contained in the database. Diagnoses in this database are coded using the *International Classification of Disease-10*^*th*^ *Revision-Clinical Modification* (ICD-10-CM) nomenclature. Patients were not invited to comment on the study design and were not consulted to develop patient-relevant outcomes or interpret the results. Patients were not invited to contribute to the writing or editing of this document for readability or accuracy.

### Study cohort

**Fig 1:**
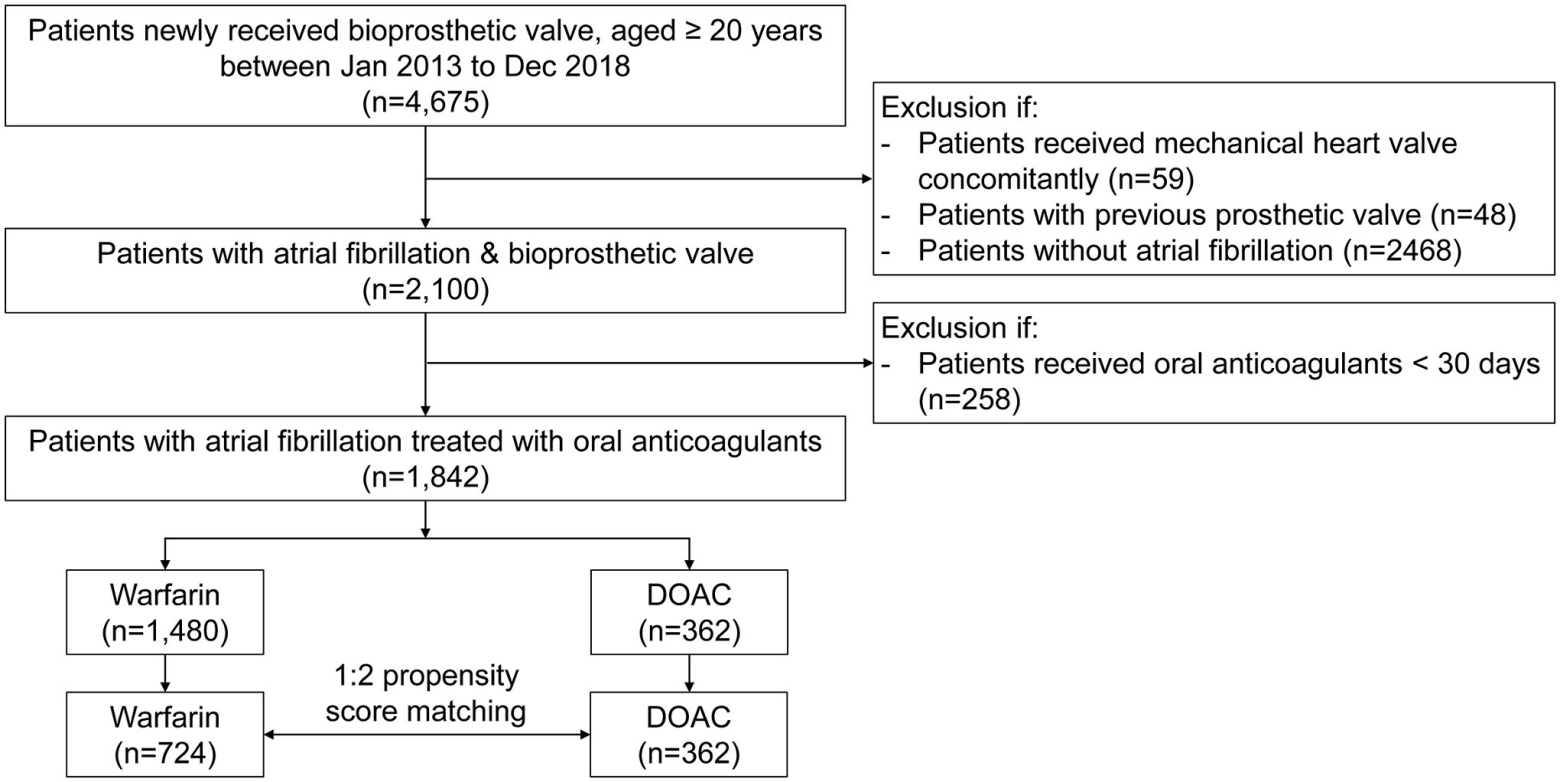
Study flow of the participant selection. DOAC, non-vitamin K direct oral anticoagulants.

Using codes for medical devices and surgical procedures (S1 Table), we included adult patients who had BPHV newly implanted between January 2013 to December 2018. Patients who had concomitant mechanical heart valve or had previous prosthetic valvular replacement (ICD-10-CM code Z952-954) were excluded. Thereafter, we identified AF patients (ICD-10-CM code I480-484, I489) who had oral anticoagulants prescribed for at least 30 days, either DOAC or warfarin. We initially constructed the entire cohort with AF patients who used either the DOAC or warfarin, and then, the patients were propensity score-matched in a 1:2 manner. The detailed patient enrollment flow is presented in Fig 1.

### Defining the comorbidities and outcomes

Comorbidities were defined using the ICD-10-CM codes (S2 Table). The use of antiplatelet, the type of heart valve used for replacement, and the type of replacement operation were identified using the code of prescription, procedure, and surgery. The CHA_2_DS_2_-VASc score was calculated for each patient by using the known criteria [1].

We evaluated 4 major clinical outcomes (S3 Table) to analyze the effectiveness and safety of DOAC versus warfarin [8,13]. The primary outcome was a composite of hospitalization under the diagnosis of ISSE after three weeks of anticoagulation. For important secondary outcomes, major bleeding (intracranial hemorrhage + gastrointestinal bleeding), all-cause death, and the net clinical outcome–a composite of ISSE, major bleeding, and all-cause death–were also defined by the ICD-10-CM codes. Follow-up was initiated from the date of the index treatment by either the DOAC or warfarin and ended with either the occurrence of a clinical event or the end of the study period (December 31, 2018), whichever came first. The nationwide insurance coverage for DOAC use was expanded from July 2015; therefore, the difference in follow-up duration existed between the two groups. We performed an analysis with the restriction of the follow-up period to 18-month in a propensity score-matched cohort to adjust the difference.

### Sensitivity and subgroup analyses

Sensitivity analysis was performed using a multivariable Cox-proportional regression method with the entire cohort, adjusting for possible variables that could further affect the outcomes, such as antiplatelet and types of BPHV. Additionally, we performed a sensitivity analysis for overall events during the entire follow-up duration in the propensity score-matched cohort.

To test for possible interaction between the use of DOAC and the specific subgroups, subgroup analyses were performed on the basis of several baseline characteristics, including age, sex, CHA_2_DS_2_-VASc score. In the age subgroup analysis, we categorized patients into three groups as follows: age of <65, 65–74, and ≥75 years old. Regarding the CHA_2_DS_2_-VASc score, we divided the total study population into two groups; 0~2 and ≥3 points.

### Statistical analysis

We used the propensity score matching method to reduce the effect of treatment-selection bias and the potential confounding factors between the two treatment groups (DOAC versus warfarin). The propensity for each treatment group was generated using a logistic regression method including the entire clinical variables: age, sex, hypertension, diabetes mellitus, dyslipidemia, heart failure, vascular disease, chronic kidney disease, chronic obstructive lung disease, CHA_2_DS_2_-VASc score, use of antiplatelet, and any previous history of stroke, intracranial hemorrhage, gastrointestinal bleeding. The balance of each covariate between the two treatment groups was evaluated using the absolute standardized difference. An absolute standardized difference <10% was considered to balance each covariate between the two treatment groups. We matched the DOAC and warfarin group in a 1:2 ratio.

The cumulative event rates between the DOAC and warfarin groups were demonstrated using Kaplan-Meier censoring estimates and compared using the Prentice-Wilcoxon test. Event rates were described as the number of events per 100 person-years. The risk for 4 clinical outcomes in the two treatment groups was presented with hazard ratios (HR) and the corresponding 95% confidence intervals (CI) calculated by Cox proportional hazard models. For the sensitivity analysis and subgroup analyses, we used a multivariable Cox proportional hazard regression method adjusting for all available factors. All *P*-values were two-sided and a p-value <0.05 was considered statistically significant. Statistical analyses were performed using SAS version 9.3 (SAS Institute, Cary, NC, USA).

## Results

### Baseline characteristics of the study population

We identified 1,842 oral anticoagulant users with AF and BPHV between 2013 and 2018. The study population was distributed into two groups; 362 patients who have dispensed DOAC and 1,480 warfarin users. In the entire cohort, the DOAC group was older, and the prevalence of comorbidities was higher than the warfarin group (S4 Table).

After 1:2 propensity score-matching, a total cohort of 1,086 patients was constructed, and the baseline characteristics of the cohort are presented in Table 1. All differences of baseline covariates were less than the absolute standardized difference of 10%, with well-distributed individual propensity scores and balanced assessments after matching (S1 Fig). The mean age was 78.9±6.8 years old, and 489 (45.0%) patients were male. The mean CHA_2_DS_2_-VASc score was 4.7±1.4. The aortic valve was replaced with a BPHV in 700 (64.5%) and mitral valve in 461 (42.4%) patients. The warfarin group received more surgical BPHV than the DOAC group (97.2% vs. 77.1%, *P*<0.001). The time interval between the valve replacement and the study enrollment was longer in the DOAC group (S5 Table).

**Table 1:** Baseline characteristics of patients with AF and BPHV according to oral anticoagulant.

	Warfarin (n = 724)	DOAC (n = 362)	P-value	ASD (%)
**Age, years**	78.9±6.6	79.0±7.0	0.960	0.93
**<65**	14 (1.9%)	10 (2.8%)	0.958	
**65–74**	151 (20.9%)	74 (20.4%)		
**≥75**	559 (77.2%)	278 (76.8%)		
**Male**	325 (44.9%)	164 (45.3%)	0.233	-0.83
**Comorbidities**				
**Hypertension**	643 (88.8%)	324 (89.5%)	1.000	2.22
**Diabetes**	359 (49.6%)	181 (50.0%)	0.579	0.83
**Dyslipidemia**	611 (84.4%)	306 (84.5%)	0.800	0.38
**Heart failure**	464 (64.1%)	236 (65.2%)	0.845	2.31
**Vascular disease**	192 (26.5%)	101 (27.9%)	0.689	3.10
**Chronic kidney disease**	68 (9.4%)	36 (9.9%)	0.431	1.87
**End-stage renal disease**	4 (0.6%)	2 (0.6%)	1.000	0.00
**COPD**	213 (29.4%)	113 (31.2%)	0.469	3.90
**Previous stroke**	35 (4.8%)	19 (5.3%)	0.706	1.89
**Previous ICH**	149 (20.6%)	89 (24.6%)	0.152	9.58
**Previous GI bleeding**	26 (3.6%)	12 (3.3%)	0.225	-1.51
**CHA**_**2**_ **DS**_**2**_ **-VASc score**	4.7±1.4	4.7±1.4	0.750	2.12
**0–2**	42 (5.8%)	22 (6.1%)	0.964	
**≥3**	682 (94.2%)	340 (93.9%)		
**Antiplatelet**	424 (58.6%)	211 (58.3%)	0.493	-0.56
**Replacement valve**			0.212	
**Aortic valve**	451 (55.6%)	249 (62.9%)		
**Mitral valve**	328 (40.4%)	133 (33.6%)		
Others*	32 (3.9%)	14 (3.5%)		-
**Replacement type**			<0.001	
**Surgical**	704 (97.2%)	279 (77.1%)		
**Transcatheter**	20 (4.1%)	83 (22.9%)		

Values are mean ± standard deviation or %.

*Other valvular disease included tricuspid valve and pulmonary valve.

Abbreviation: AF, atrial fibrillation ASD, absolute standardized difference; BPHV, bioprosthetic heart valve; COPD, chronic obstructive pulmonary disease; GI, gastrointestinal; ICH, intracranial hemorrhage; DOAC, non-vitamin K antagonist direct oral anticoagulant.

### Clinical outcomes in patients with AF and BPHV

**Fig 2:**
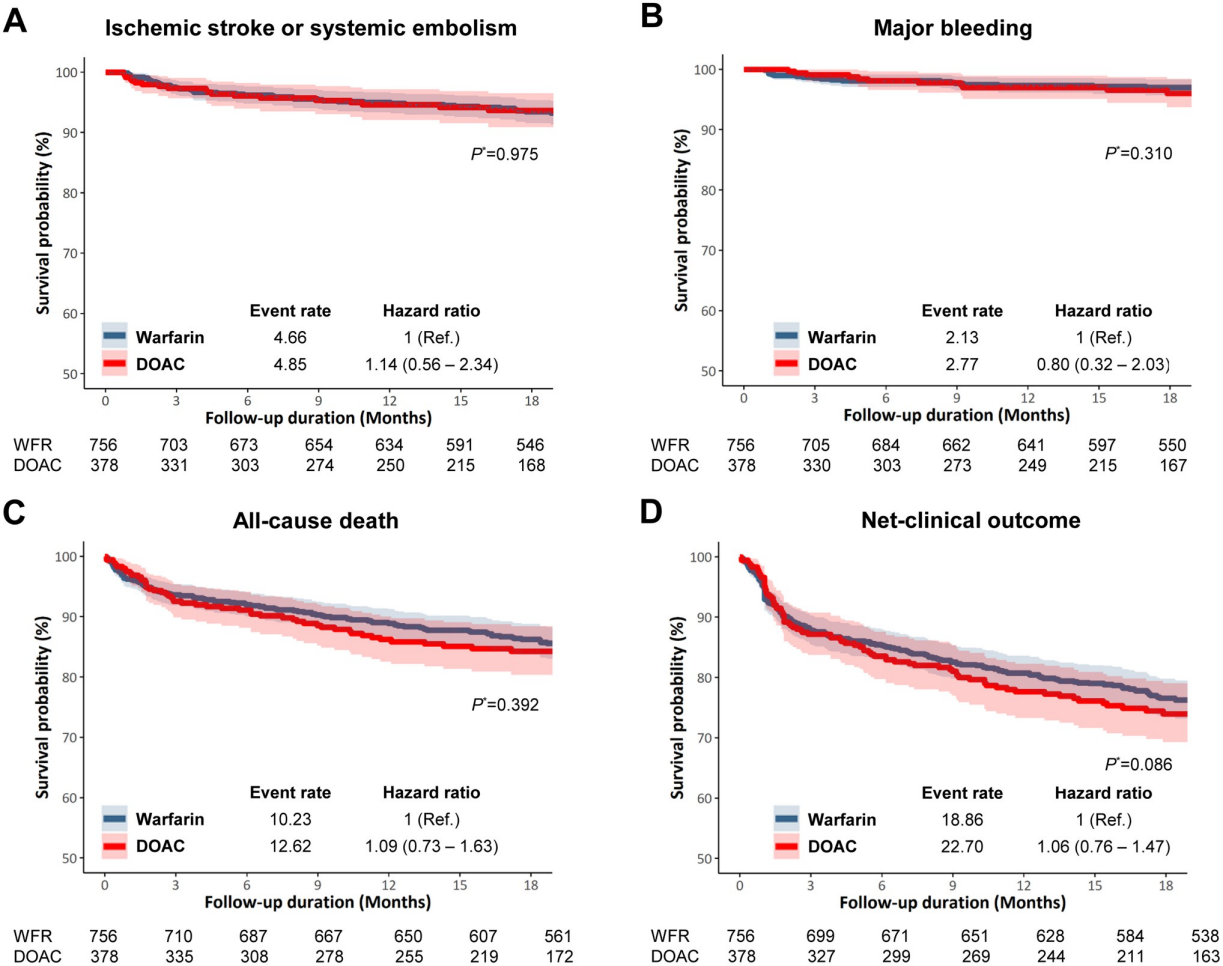
Cumulative incidence curves, event rates and hazard ratios of clinical outcomes in DOAC versus warfarin users. **(A)** Ischemic stroke or systemic embolism event. **(B)** Major bleeding, defined as either intracranial hemorrhage or gastrointestinal bleeding. **(C)** All-cause death. **(D)** Net clinical outcome, defined as the composite of ischemic stroke, systemic embolism event, major bleeding, and all-cause death. *P-value by Prentice-Wilcoxon test. DOAC, non-vitamin K direct oral anticoagulants.

During a mean 1.2 years follow-up, there were 19 ISSE events in the DOAC and 43 in the warfarin group. The event rate for ISSE was 4.85 per 100 person-years for DOAC versus 4.66 per 100 person-years for the warfarin group (Table 2). The risk of ISSE was not different (HR 1.14, 95% CI 0.56–2.34, *P* = 0.715; Table 2, Fig 2A).

**Table 2:** Event numbers, incidence rates, and hazard ratios of 4 clinical outcomes in DOAC versus warfarin groups.

GROUP	N	Event	Duration (years)	Event Rate*	Hazard Ratios (95% CI)	P-value
**Ischemic stroke + Systemic embolism**						
**Warfarin**	724	43	922.7	4.66	1(Ref.)	
**DOAC**	362	19	391.7	4.85	1.14 (0.56–2.34)	0.715
**Major bleeding**						
**Warfarin**	724	20	941.2	2.13	1(Ref.)	
**DOAC**	362	11	397.5	2.77	0.80 (0.32–2.03)	0.638
**All-cause death**						
**Warfarin**	724	98	957.7	10.23	1(Ref.)	
**DOAC**	362	51	404.0	12.62	1.09 (0.73–1.63)	0.680
**Net clinical outcome**						
**Warfarin**	724	167	885.3	18.86	1(Ref.)	
**DOAC**	362	84	370.1	22.70	1.06 (0.76–1.47)	0.739

*Incidence rate is presented as per 100 person-years.

Abbreviation: AF, atrial fibrillation; BPHV, bioprosthetic heart valve; CI, confidence interval; DOAC, non-vitamin K antagonist direct oral anticoagulant.

As for the safety outcomes, major bleeding occurred at an event rate of 2.77 per 100 person-years for DOAC and 2.13 per 100 person-years for warfarin group, which was also not statistically different (HR 0.80, 95% CI 0.32–2.03; Table 2, Fig 2B). The event rate for all-cause death was 12.62 per 100 person-years for the DOAC and 10.23 per 100 person-years for warfarin group, which was again similar between the two groups (HR 1.09, 95% CI 0.73–1.63; Table 2, Fig 2C). As for the net clinical outcome, defined as a composite of ISSE, major bleeding, and all-cause death, the DOAC group was also not different compared to the warfarin group (HR 1.06, 95% CI 0.76–1.47; Table 2, Fig 2D).

### Sensitivity and subgroup analysis

Consistent results were demonstrated by the sensitivity analysis that further adjusted for other covariates. Again, the DOAC group was similar to warfarin in terms of the 4 clinical outcomes (S6 Table). When we also analyzed the overall events over the entire duration of follow-up, the results were also consistent with the main analysis (S7 Table).

The crude incidences and HR of endpoints in the DOAC and the warfarin users by age, sex, and CHA_2_DS_2_-VASc score subgroups are presented (S8 Table). There were no significant interactions with respect to ISSE, major bleeding, all-cause death, and the net-clinical outcome between the treatment allocation and the various subgroups.

## Discussion

In this study, we analyzed the effectiveness and safety of DOAC for ISSE prevention in AF patients with BPHV compared to warfarin. In the propensity score-matched cohort, the event rates of ISSE, major bleeding, all-cause death, and the net clinical outcome were similar between the two groups. Our main finding is that DOAC has comparable effectiveness and safety as warfarin for ISSE prevention in AF patients with BPHV.

Recently, the use of BPHV has become more and more popular than a mechanical valve [14]. Acquired valvular heart disease increases with age [15,16], and guidelines recommend the use of BPHV than mechanical valve for valvular surgery in elderly patients (>65 years old for aortic valve or >70 years old for mitral valve). Moreover, the development of transcatheter aortic valve replacement has allowed the surgeons to consider a BPHV at a younger age more actively, with the option of the valve-in-valve procedure later on [17]. Although data on the epidemiology of patients who have both AF and valvular heart disease is limited, the prevalence of AF increases dramatically with age [18,19]. Therefore, AF patients with BPHV will inevitably increase in elderly patients, with physicians encountering these patients more and more frequently and facing the clinical enigma of what drugs to use to prevent ISSE in these patients.

### DOAC use in AF patients with BPHV

To date, there is limited evidence to support the use of DOAC instead of warfarin in AF patients with BPHV. Recently, subclinical leaflet thickening and/or thrombosis has been demonstrated in BPHV [20], suggesting the need for effective measures to prevent thromboembolic events. Although the risk of thromboembolic events may be reduced by warfarin in patients with BPHV [10,21], a recent randomized trial has shown disappointing results with rivaroxaban in patients with the transcatheter aortic valve [22]. The study sought to investigate the impact of rivaroxaban 10mg for preventing thromboembolic events after transcatheter aortic valve replacement without established indication of oral anticoagulation, such as AF. As such, it is difficult to extrapolate the results of this trial to explain our study results. In those with mechanical heart valves, dabigatran has also been shown to be inferior to warfarin [23]. These findings demonstrate that non-inferior or superior results of DOAC over other anticoagulants or antiplatelets in nonvalvular AF do not always warrant the same efficacy and safety in other groups, especially in those with valvular heart disease or prosthetic heart valves.

Although DOAC seemed to be non-inferior to warfarin in post-hoc analysis of pivotal randomized trials in patients with AF [11,24], these results should be taken cautiously because of the limited number of BPHV patients and clinical events for each group. Recently, the evidence for non-inferiority of rivaroxaban versus warfarin has been updated from the RIVER trial [12]. However, this study exclusively included patients with AF and bioprosthetic mitral valve replacement. The patients were younger (median 59.3 years) and had a lower risk of stroke and bleeding (CHA_2_DS_2_-VASc score = 2.6±1.4, HAS-BLED score = 1.6±0.9) than our study because the RIVER trial excluded high-risk patients. Another randomized trial compared edoxaban and warfarin in patients after surgical bioprosthetic valve replacement or valve repair, regardless of the prevalence of AF [25]. This study also showed the inferiority of edoxaban for preventing thromboembolism during the first 3 months. However, the purpose of the study was to investigate the early postoperative outcomes, which was different from ours, and only 62% of patients had AF. Our results demonstrate that DOAC would be at least as effective as warfarin in preventing embolic events and also comparable in terms of safety for AF patients with BPHV in real-world practice. Additionally, these results were consistent throughout the various subgroups analyzed as well. The strengths of our analysis are that it is the largest observational cohort to date that encompasses AF patients with any BPHV and that the results reflect the safety and effectiveness of DOAC within a real-world, high-risk setting in these patients. It also calls for future large randomized trials to resolve the clinical enigma in this population that is expected to grow significantly in the future.

### Current trend of oral anticoagulation for AF patients with BPHV

To our knowledge, our study is the largest cohort of AF patients with BPHV who received oral anticoagulants for ISSE prevention [10]. Recent expert consensus statement carefully categorized the patients with BPHV to the group with valvular heart disease that could use DOAC instead of warfarin for ISSE prevention [6]. However, despite these statements, our results also showed that clinicians still prefer warfarin over DOAC for AF patients with BPHV in real-world practice; approximately 80% of patients received warfarin in the entire cohort. Interestingly, the DOAC group was older and had a higher proportion of comorbidities (especially higher prevalence of hypertension, heart failure, and previous intracranial hemorrhage; S4 Table) compared to the warfarin group. This prescription pattern may be because DOAC show better bleeding outcomes than warfarin in Asian patients [26]. Therefore, the physicians in Korea are likely to prescribe DOAC for older and fragile groups.

Although not every DOAC have appropriate antidotes compared to warfarin, it is well known that DOAC does not need monitoring of the therapeutic concentration, is stable in terms of therapeutic serum levels, and leads to better compliance than warfarin. Because our analysis is based on a cohort study, the results should be taken as hypothesis-generating, however, if proven in future prospective studies or trials, the results would definitely have a large impact on the prescription pattern of these drugs.

### Limitations

First, diagnoses were defined by ICD-10 codes, which carry the inherent limitation of coding errors. However, the definition of AF, comorbidities, and clinical outcomes have been validated in several previous studies [8,12,27]. Second, although we performed propensity score matching and the two treatment groups well balanced, hidden confounding factors might still exist. In the procedure types of BPHV, the patients with transcatheter aortic valve replacement were higher in the DOAC group. Furthermore, the time interval between the valve replacement and the study enrollment was longer in DOAC group, which may be a confounding factor of the study results. However, the outcomes were similar between the two groups even after adjusting these differences. Third, we could not evaluate the treatment quality of warfarin, an inherent limitation of the claims data. Lastly, as for the types of BPHV, the patients who underwent transcatheter aortic valve replacement were higher in the DOAC group. Although it would have been most ideal to adjust this perfectly, it was impossible because introducing new procedures or drugs (transcatheter aortic valve replacement and DOAC in this case) greatly depends on their induction into the medical insurance system.

## Conclusions

This nationwide population-based study suggests that DOAC has similar effectiveness and safety as warfarin in AF patients with BPHV for the prevention of ISSE. Therefore, DOAC could be an acceptable alternative to warfarin for ISSE prevention in these patients.

## Supporting information

S1 Fig Distribution of propensity scores in DOAC and warfarin groups before and after propensity score matching. (DOCX)

S1 Table Definitions of prosthetic heart valve. (DOCX)

S2 Table Definitions of covariates. (DOCX)

S3 Table Definitions of clinical outcomes. (DOCX)

S4 Table Entire cohort of patients with AF and BPHV according to oral anticoagulant. (DOCX)

S5 Table The time interval between valve replacement and enrollment in AF patients with BPHV. (DOCX)

S6 Table Event numbers, incidence rates, and hazard ratios of 4 clinical outcomes using multivariate Cox regression analysis in DOAC versus warfarin in AF patients with BPHV. (DOCX)

S7 Table Sensitivity analysis during the whole follow-up period. (DOCX)

S8 Table Number of events, crude event rates and hazard ratios according to various subgroups in AF patients with BPHV. (DOCX)
