# Supplementary material for: Effectiveness and safety of non-vitamin K direct oral anticoagulants in atrial fibrillation patients with bioprosthetic valve
Source: PLoS One. 2022 Jun 14;17(6):e0268113. doi: 10.1371/journal.pone.0268113 (PMC9197068; doi:10.1371/journal.pone.0268113)

**Supplementary Figure 1.** **Distribution of propensity scores in DOAC and warfarin groups before and after propensity score matching**
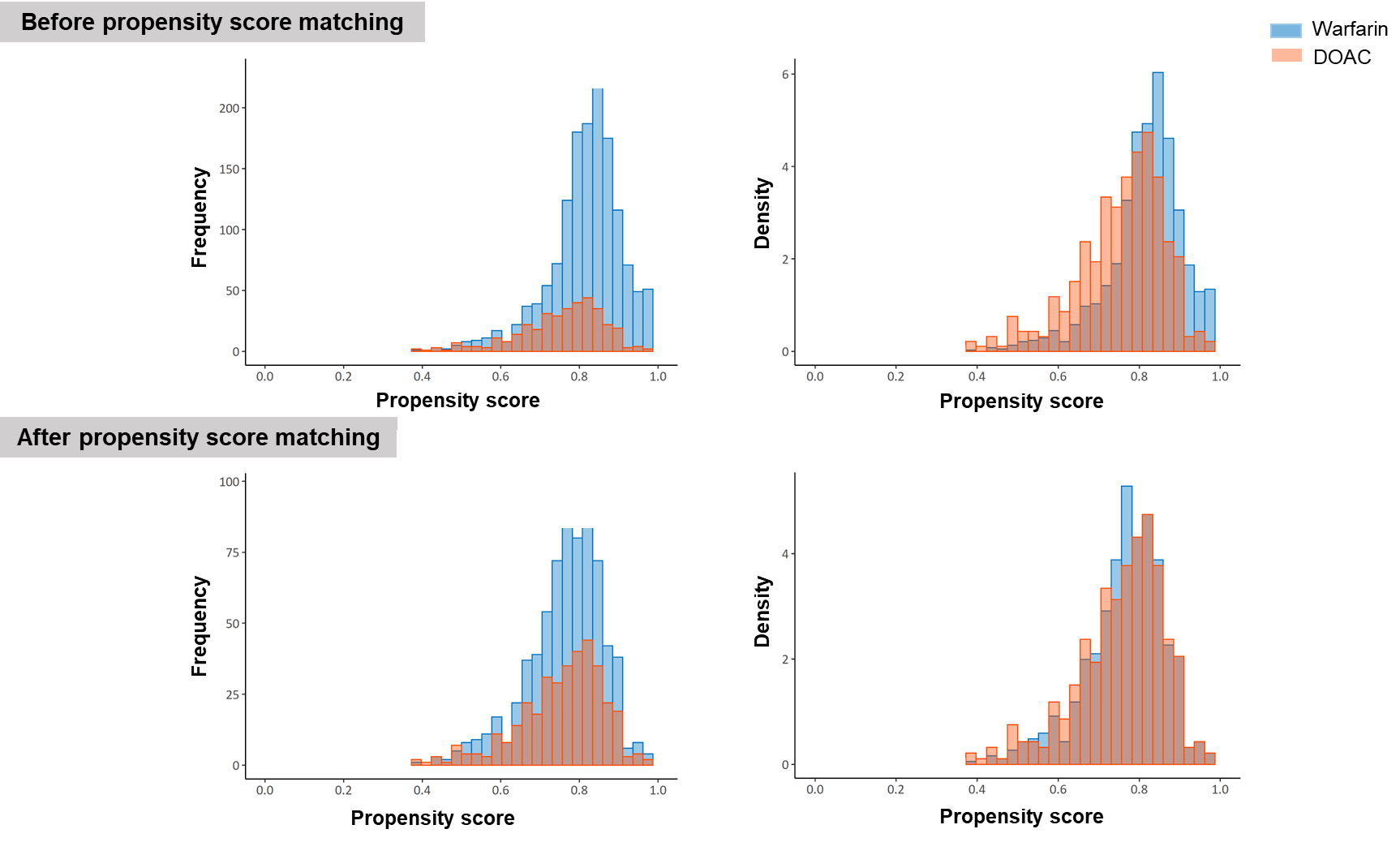

Supplement: S1 Fig — (DOCX) [file pone.0268113.s001.docx]
