# Supplementary material for: Effectiveness and safety of non-vitamin K direct oral anticoagulants in atrial fibrillation patients with bioprosthetic valve
Source: PLoS One. 2022 Jun 14;17(6):e0268113. doi: 10.1371/journal.pone.0268113 (PMC9197068; doi:10.1371/journal.pone.0268113)
Supplement: S1 Table — (DOCX) [file pone.0268113.s002.docx]

**Supplementary Table 1.** **Definitions of prosthetic heart valve**

| **Product name** | **Product code** | **Manufacture company** |
| --- | --- | --- |
| **Surgical bioprosthetic heart valve** | |  |
| Carpentier Edwards Perimount valve | G2001002 | EDWARDS LIFESCIENCES |
| Hancock II valve | G2001003 | MEDTRONIC, INC. |
| Soprano Pericardial Heart valve | G2001034 | SORIN GROUP ITALIA S.R.L |
| Carpentier Edwards Perimount Magna TFX valve | G2001102 | EDWARDS LIFESCIENCES |
| Mosaic Tissue valve | G2001103 | MEDTRONIC HEART VALVES |
| SJM Epic valve | G2001121 | ST.JUDE MEDICAL |
| Pericarbon More Pericardial Heart valve | G2001134 | SORIN GROUP ITALIA S.R.L |
| Avalus Bioprosthesis | G2001203 | MEDTRONIC, INC. |
| Epic Supra valve | G2001221 | ST.JUDE MEDICAL |
| Mitroflow Aortic Pericardial Heart valve, Crown Prtaortic Pericardial Heart valve | G2001234 | LIVANOVA CANADA CORP. |
| Intuity Elite valve system | G2301002 | EDWARDS LIFESCIENCES |
| **Transcatheteric bioprosthetic heart valve** | |  |
| Edwards Sapien XT/3 Transfemoral KIT | G2201002 | EDWARDS LIFESCIENCES, LLC |
| Core valve system | G2201003 | MEDTRONIC MEXICO S.DE.R.L.DE C.V/MEDTRONIC IRELAND |
| Portico Transcatheter Heart valve | G2201021 | ST.JUDE MEDICAL |
| Lotus valve system | G2201025 | SADRA MEDICAL INC./BOSTON SCIENTIFIC IRELAND LTD. |
| **Surgical mechanical heart valve** | |  |
| Med Hall valve | G2011003 | MEDTRONIC, INC. |
| SJM Masters Series valve | G2011007 | ST.JUDE MEDICAL |
| ON-X Mechanical Heart valve | G2011011 | MEDICAL CARBON RESEARCH INSTITUTE |
| Bicarbon Heart valve | G2011012 | SORIN BIOMEDICA |
| SJM Regent Mechanical Heart valve | G2011021 | ST.JUDE MEDICAL |
| Bcarbon Mechanical Heart valve | G2011034 | SORIN GROUP ITALIA S.R.L |
| ATS Heart valve | G2011129 | MEDTRONIC, INC. |
