# Supplementary material for: Effectiveness and safety of non-vitamin K direct oral anticoagulants in atrial fibrillation patients with bioprosthetic valve
Source: PLoS One. 2022 Jun 14;17(6):e0268113. doi: 10.1371/journal.pone.0268113 (PMC9197068; doi:10.1371/journal.pone.0268113)
Supplement: S2 Table — (DOCX) [file pone.0268113.s003.docx]

**Supplementary Table 2.** **Definitions of covariates**

| **Diagnosis** | **ICD-10-CM code and definition** | **Diagnostic definition** |
| --- | --- | --- |
| **Atrial fibrillation** | I480-484, I489 | Admission event or outpatient department visit ≥1 |
| **Hypertension** | I10-I13, I15; and minimum 1 prescription of anti-hypertensive drug (thiazide, loop diuretics, aldosterone antagonist, alpha-/beta-blocker, calcium-channel blocker, angiotensin-converting enzyme inhibitor, angiotensin II receptor blocker). | Admission event ≥1 or outpatient department visit ≥2 |
| **Diabetes mellitus** | E11-E14; and minimum 1 prescription of anti-diabetic drugs (sulfonylureas, metformin, meglitinides, thiazolidinediones, dipeptidyl peptidase-4 inhibitors, α-glucosidase inhibitors, and insulin). | Admission event ≥1 or outpatient department visit ≥2 |
| **Dyslipidemia** | E78 | Admission event or outpatient department visit ≥1 |
| **Congestive heart failure** | I50 | Admission event or outpatient department visit ≥1 |
| **Vascular disease** |  |  |
| **Prior MI** | I21, I22 | Admission event or outpatient department visit ≥1 |
| **PAD** | I70, I73 | Admission event or outpatient department visit ≥2 |
| **COPD** | J41-44 | Admission event or outpatient department visit ≥1 |
| **Chronic kidney disease** | N18 | Admission event or outpatient department visit ≥1 |
| **End stage renal disease** | N185, Z49 | Dialysis ≥2 |
| **Liver cirrhosis** | K70-77 | Admission event or outpatient department visit ≥1 |
| **Ischemic stroke** | I63, I64 | Admission event ≥1 and brain imaging (CT or MRI) ≥1 |
| **Intracranial hemorrhage** | I60-62 | Admission event ≥1 or RBC transfusion event ≥1 |
| **GI bleeding** | K22.6, K25.0, K25.2, K25.4, K25.6, K26.0, K26.2, K26.4, K26.6, K27.0, K27.2, K27.4, K27.6, K28.0, K28.2, K28.4, K28.6, K29.0, K62.5, K92.0, K92.1, K92.2 | Admission event ≥1 and RBC transfusion event ≥1 |

Abbreviation: COPD, chronic obstructive pulmonary disease; GI, gastrointestinal; MI, myocardial infarction; PAD, peripheral artery disease.
