# Supplementary material for: Effectiveness and safety of non-vitamin K direct oral anticoagulants in atrial fibrillation patients with bioprosthetic valve
Source: PLoS One. 2022 Jun 14;17(6):e0268113. doi: 10.1371/journal.pone.0268113 (PMC9197068; doi:10.1371/journal.pone.0268113)
Supplement: S3 Table — (DOCX) [file pone.0268113.s004.docx]

**Supplementary Table 3.** **Definitions of clinical outcomes**

| **Clinical outcomes** | **ICD-10-CM code and definition** | **Diagnostic definition** |
| --- | --- | --- |
| **Ischemic stroke** | I63, I64 | Admission event ≥1 and brain imaging (CT or MRI) ≥1 |
| **Systemic embolism** | N280, D735, K550, I740-745, 748,749 | Admission event ≥1 |
| **Intracranial hemorrhage** | I60-62 | Admission event ≥1 or RBC transfusion event ≥1 |
| **Gastrointestinal bleeding** | K22.6, K25.0, K25.2, K25.4, K25.6, K26.0, K26.2, K26.4, K26.6, K27.0, K27.2, K27.4, K27.6, K28.0, K28.2, K28.4, K28.6, K29.0, K62.5, K92.0, K92.1, K92.2 | Admission event ≥1 and RBC transfusion event ≥1 |
| **Net-clinical outcome** | Ischemic stroke, systemic embolism, intracranial hemorrhage, gastrointestinal bleeding or all-cause death | Ischemic stroke, Admission event ≥1 and brain imaging (CT or MRI) ≥1  Systemic embolism, admission event ≥1  ICH, admission event ≥1 or RBC transfusion event ≥1  GI bleeding, admission event ≥1 and RBC transfusion event ≥1 |

Abbreviation: GI, gastrointestinal; ICH, intracranial hemorrhage
