# Supplementary material for: Effectiveness and safety of non-vitamin K direct oral anticoagulants in atrial fibrillation patients with bioprosthetic valve
Source: PLoS One. 2022 Jun 14;17(6):e0268113. doi: 10.1371/journal.pone.0268113 (PMC9197068; doi:10.1371/journal.pone.0268113)
Supplement: S4 Table — (DOCX) [file pone.0268113.s005.docx]

**Supplementary Table 4.** **Entire cohort of patients with AF and BPHV according to oral anticoagulant.**

|  | **Warfarin**  **(n=1480)** | **DOAC**  **(n=362)** | **P-value** |
| --- | --- | --- | --- |
| **Age, years** | 75.9±9.4 | 79.0±7.0 | <0.001 |
| **<65** | 104 (7.0%) | 10 (2.8%) | <0.001 |
| **65-74** | 408 (27.6%) | 74 (20.4%) |  |
| **≥75** | 968 (65.4%) | 278 (76.8%) |  |
| **Male** | 668 (45.1%) | 164 (45.3%) | 0.954 |
| **Comorbidities** |  |  |  |
| **Hypertension** | 1,211 (81.8%) | 324 (89.5%) | <0.001 |
| **Diabetes** | 705 (47.6%) | 181 (50.0%) | 0.420 |
| **Dyslipidemia** | 1,124 (76.0%) | 306 (84.5%) | 0.083 |
| **Heart failure** | 838 (56.6%) | 236 (65.2%) | 0.003 |
| **Vascular disease** | 310 (21.0%) | 101 (27.9%) | 0.004 |
| **Chronic kidney disease** | 148 (10.0%) | 36 (9.9%) | 0.975 |
| **End-stage renal disease** | 51 (3.5%) | 2 (0.6%) | 0.003 |
| **COPD** | 443 (29.9%) | 113 (31.2%) | 0.634 |
| **Previous stroke** | 86 (5.8%) | 19 (5.3%) | 0.679 |
| **Previous ICH** | 190 (12.8%) | 89 (24.6%) | <0.001 |
| **Previous GI bleeding** | 43 (2.9%) | 12 (3.3%) | 0.682 |
| **CHA_2_DS_2_-VASc score** | 4.3±1.5 | 4.7±1.4 | <0.001 |
| **0-2** | 168 (11.4%) | 22 (6.1%) | 0.003 |
| **≥3** | 1,312 (98.6%) | 340 (93.9%) |  |
| **Antiplatelet** | 835 (56.4%) | 211 (58.3%) | 0.520 |
| **Replacement valve** |  |  | 0.007 |
| **Aortic valve** | 925 (55.2%) | 258 (63.6%) |  |
| **Mitral valve** | 661 (39.4%) | 134 (33.0%) |  |
| **Others**^*^ | 90 (5.4%) | 14 (3.5%) |  |
| **Replacement type** |  |  | <0.001 |
| **Surgical** | 1,446 (97.7%) | 279 (77.1%) |  |
| **Transcatheter** | 34 (2.3%) | 83 (22.9%) |  |

Values are mean ± standard deviation or %.

^*^Other valvular disease included tricuspid valve and pulmonary valve.

Abbreviation: AF, atrial fibrillation ASD, absolute standardized difference; BPHV, bioprosthetic heart valve; COPD, chronic obstructive pulmonary disease; GI, gastrointestinal; ICH, intracranial hemorrhage; DOAC, non-vitamin K direct oral anticoagulant.
