# Supplementary material for: Effectiveness and safety of non-vitamin K direct oral anticoagulants in atrial fibrillation patients with bioprosthetic valve
Source: PLoS One. 2022 Jun 14;17(6):e0268113. doi: 10.1371/journal.pone.0268113 (PMC9197068; doi:10.1371/journal.pone.0268113)
Supplement: S5 Table — (DOCX) [file pone.0268113.s006.docx]

**Supplementary Table 5. The time interval between valve replacement and enrollment in AF patients with BPHV**

| **Time interval (month)** | **Warfarin**  **(n=724)** | **DOAC**  **(n=362)** | **Total**  **(n=1086)** |
| --- | --- | --- | --- |
| **< 1** | 706 (97.5%) | 165 (45.6%) | 871 (80.2%) |
| **1 to < 6** | 14 (1.9%) | 48 (13.3%) | 62 (5.7%) |
| **6 to < 12** | 3 (0.4%) | 26 (7.2%) | 29 (2.7%) |
| **≥ 12** | 1 (0.1%) | 123 (34.0%) | 124 (11.4%) |

Abbreviation: AF, atrial fibrillation; BPHV, bioprosthetic heart valve; DOAC, non-vitamin K direct oral anticoagulant.
