# Supplementary material for: Effectiveness and safety of non-vitamin K direct oral anticoagulants in atrial fibrillation patients with bioprosthetic valve
Source: PLoS One. 2022 Jun 14;17(6):e0268113. doi: 10.1371/journal.pone.0268113 (PMC9197068; doi:10.1371/journal.pone.0268113)
Supplement: S6 Table — (DOCX) [file pone.0268113.s007.docx]

**Supplementary Table 6.** **Event numbers, incidence rates, and hazard ratios of 4 clinical outcomes using multivariate Cox regression analysis in DOAC versus warfarin in AF patients with BPHV**

| **GROUP** | **N** | **Event** | **Duration (years)** | **Incidence Rate^*^** | **Adjusted Hazard Ratios^†^**  **(95% CI)** | **P-value** |
| --- | --- | --- | --- | --- | --- | --- |
| **Ischemic stroke + Systemic embolism** | | | | | | |
| **Warfarin** | 1480 | 77 | 1910.0 | 4.03 | 1(Ref.) |  |
| **DOAC** | 362 | 19 | 391.7 | 4.85 | 1.11 (0.64 - 1.91) | 0.723 |
| **Major bleeding** | | | | | | |
| **Warfarin** | 1480 | 43 | 1939.6 | 2.22 | 1(Ref.) |  |
| **DOAC** | 362 | 11 | 397.6 | 2.77 | 1.36 (0.69 - 2.70) | 0.374 |
| **All-cause death** | | | | | | |
| **Warfarin** | 1480 | 187 | 1970.2 | 9.49 | 1(Ref.) |  |
| **DOAC** | 362 | 51 | 404.0 | 12.62 | 1.02 (0.74 - 1.41) | 0.909 |
| **Net clinical outcome** | | | | | | |
| **Warfarin** | 1480 | 314 | 1838.2 | 17.08 | 1(Ref.) |  |
| **DOAC** | 362 | 84 | 370.1 | 22.70 | 1.08 (0.84 - 1.38) | 0.565 |

*Incidence rate is presented as per 100 person-years.

**^†^**Adjusted with all available risk factors

Abbreviation: AF, atrial fibrillation; BPHV, bioprosthetic heart valve; CI, confidence interval; DOAC, non-vitamin K direct oral anticoagulant.
