# Supplementary material for: Effectiveness and safety of non-vitamin K direct oral anticoagulants in atrial fibrillation patients with bioprosthetic valve
Source: PLoS One. 2022 Jun 14;17(6):e0268113. doi: 10.1371/journal.pone.0268113 (PMC9197068; doi:10.1371/journal.pone.0268113)
Supplement: S7 Table — (DOCX) [file pone.0268113.s008.docx]

**Supplementary Table 7. Sensitivity analysis during the whole follow-up period**

| **GROUP** | **N** | **Event** | **Duration (years)** | **Incidence Rate^*^** | **Hazard Ratios**  **(95% CI)** | **P-value** |
| --- | --- | --- | --- | --- | --- | --- |
| **Ischemic stroke + Systemic embolism** | | | | | | |
| **Warfarin** | 724 | 62 | 1911.7 | 3.243 | 1(Ref.) |  |
| **DOAC** | 362 | 23 | 546.0 | 4.212 | 1.13 (0.57 - 2.21) | 0.732 |
| **Major bleeding** | | | | | | |
| **Warfarin** | 724 | 30 | 1983.5 | 1.513 | 1(Ref.) |  |
| **DOAC** | 362 | 16 | 550.8 | 2.905 | 1.00 (0.42 - 2.40) | 1.000 |
| **All-cause death** | | | | | | |
| **Warfarin** | 724 | 217 | 2032.4 | 10.677 | 1(Ref.) |  |
| **DOAC** | 362 | 93 | 568.5 | 16.358 | 1.39 (0.96 - 2.00) | 0.080 |
| **Net clinical outcome** | | | | | | |
| **Warfarin** | 724 | 279 | 1822.2 | 15.311 | 1(Ref.) |  |
| **DOAC** | 3762 | 118 | 498.9 | 23.653 | 1.13 (0.83 - 1.55) | 0.430 |

*Incidence rate is presented as per 100 person-years.

Abbreviation: AF, atrial fibrillation; BPHV, bioprosthetic heart valve; CI, confidence interval; DOAC, non-vitamin K direct oral anticoagulant.
