# Supplementary material for: Effectiveness and safety of non-vitamin K direct oral anticoagulants in atrial fibrillation patients with bioprosthetic valve
Source: PLoS One. 2022 Jun 14;17(6):e0268113. doi: 10.1371/journal.pone.0268113 (PMC9197068; doi:10.1371/journal.pone.0268113)
Supplement: S8 Table — (DOCX) [file pone.0268113.s009.docx]

**Supplementary Table 8.** **Number of events, crude event rates and hazard ratios according to various subgroups in AF patients with BPHV**

| **Subgroup category** | **Treatment group** | **Total number** | **Ischemic stroke and systemic embolism** | | | | **Major bleeding** | | | | **All-cause death** | | | | **Net-clinical outcome** | | | |
| --- | --- | --- | --- | --- | --- | --- | --- | --- | --- | --- | --- | --- | --- | --- | --- | --- | --- | --- |
|  |  |  | **N** | **ER**^*^ | **HR**^†^  **(95% CI)** | ***P*^￥^** | **N** | **ER**^*^ | **HR**^†^  **(95% CI)** | ***P*^￥^** | **N** | **ER**^*^ | **HR**^†^  **(95% CI)** | ***P*^￥^** | **N** | **ER**^*^ | **HR**^†^  **(95% CI)** | ***P*^￥^** |
| **Age (years)** | | | | | | | | | | | | | | | | | | |
| **<65** | **Warfarin** | 14 | 1 | 5.9 | 1(Ref.) | 0.920 | 0 | 0.0 | 1(Ref.) | 0.765 | 0 | 0.0 | 1(Ref.) | 0.226 | 0 | 0.0 | 1(Ref.) | 0.551 |
|  | **DOAC** | 10 | 0 | 0.0 | - |  | 0 | 0.0 | - |  | 1 | 10.2 | - |  | 1 | 10.2 |  |  |
| **65-74** | **Warfarin** | 151 | 5 | 2.5 | 1(Ref.) |  | 6 | 3.0 | 1(Ref.) |  | 16 | 7.9 | 1(Ref.) |  | 16 | 7.9 | 1(Ref.) |  |
|  | **DOAC** | 74 | 3 | 3.6 | 1.91  (0.36 - 10.06) |  | 2 | 2.3 | 1.06  (0.20 - 5.56) |  | 3 | 3.4 | 0.34  (0.09 - 1.37) |  | 3 | 3.4 | 0.74  (0.31 - 1.76) |  |
| **≥75** | **Warfarin** | 559 | 37 | 5.2 | 1(Ref.) |  | 14 | 1.9 | 1(Ref.) |  | 82 | 11.1 | 1(Ref.) |  | 82 | 11.2 | 1(Ref.) |  |
|  | **DOAC** | 278 | 16 | 5.4 | 1.70  (0.63 - 2.19) |  | 9 | 3.0 | 1.06  (0.40 - 2.83) |  | 47 | 15.3 | 1.32  (0.89 - 1.96) |  | 47 | 15.3 | 1.23  (0.88 - 1.72) |  |
| **Sex** | | | | | | | | | | | | | | | | | | |
| **Male** | **Warfarin** | 325 | 14 | 3.3 | 1(Ref.) | 0.054 | 7 | 1.6 | 1(Ref.) | 0.290 | 41 | 9.3 | 1(Ref.) | 0.685 | 41 | 9.3 | 1(Ref.) | 0.103 |
|  | **DOAC** | 164 | 11 | 6.2 | 1.97  (0.81 - 4.80) |  | 6 | 3.3 | 2.11  (0.61 - 7.34) |  | 23 | 12.4 | 1.14  (0.64 - 2.03) |  | 23 | 12.4 | 1.42  (0.89 - 2.27) |  |
| **Female** | **Warfarin** | 399 | 29 | 5.9 | 1(Ref.) |  | 13 | 2.6 | 1(Ref.) |  | 57 | 11.0 | 1(Ref.) |  | 57 | 11.0 | 1(Ref.) |  |
|  | **DOAC** | 198 | 8 | 3.7 | 0.70  (0.31 - 1.57) |  | 5 | 2.3 | 0.81  (0.26 - 2.54) |  | 28 | 12.8 | 1.24  (0.76 - 2.02) |  | 28 | 12.8 | 0.97  (0.64 - 1.49) |  |
| **CHA_2_DS_2_-VASc** | | | | | | | | | | | | | | | | | | |
| **0-2** | **Warfarin** | 7 | 1 | 11.3 | 1(Ref.) | 0.987 | 0 | 0.0 | 1(Ref.) | 1.000 | 0 | 0.0 | 1(Ref.) | 0.999 | 0 | 0.0 | 1(Ref.) | 0.978 |
|  | **DOAC** | 3 | 0 | 0.0 | - |  | 0 | 0.0 | - |  | 0 | 0.0 | - |  | 0 | 0.0 | - |  |
| **≥3** | **Warfarin** | 717 | 42 | 4.6 | 1(Ref.) |  | 20 | 2.2 | 1(Ref.) |  | 98 | 10.3 | 1(Ref.) |  | 98 | 10.3 | 1(Ref.) |  |
|  | **DOAC** | 359 | 19 | 4.9 | 1.15  (0.65 – 2.06) |  | 11 | 2.8 | 1.10  (0.48 - 2.51) |  | 51 | 12.7 | 1.14  (0.79 - 1.65) |  | 51 | 12.7 | 1.12  (0.82 - 1.53) |  |

^*^Event rate, per 100 person-years

^†^Cox-proportional HR model adjusted with all-clinical variables

**^￥^**P for interaction

Abbreviation: ER, event rates; HR, hazard ratio; DOAC, non-vitamin K direct oral anticoagulant.
